# DMB-induced GSDMD-mediated pyroptosis: a novel therapeutic strategy for enhancing anti-tumor immunity

**DOI:** 10.1038/s41420-024-02248-0

**Published:** 2024-11-25

**Authors:** Jianqiao Shentu, Hanqi Lou, Shiwei Duan

**Affiliations:** Department of Clinical Medicine, Hangzhou City University, Hangzhou, Zhejiang China

**Keywords:** Drug development, Immune cell death

Recently, Professor Hao Wu’s team at Harvard University made a significant discovery, published in *Cell* [1]. They identified a specific small molecule agonist of GSDMD, quinoxaline 6,7-dichloro-2-methylsulfonyl-3-N-tert-butylaminoquinoxaline (DMB), which can directly activate GSDMD-mediated cancer cell pyroptosis. Unlike traditional pathways requiring GSDMD cleavage, DMB induces pyroptosis without this step, effectively stimulating anti-tumor immune responses with minimal toxicity. This novel strategy expands the therapeutic potential of pyroptosis in cancer treatment and highlights promising directions for future clinical applications (Fig. 1).

**Fig. 1:**
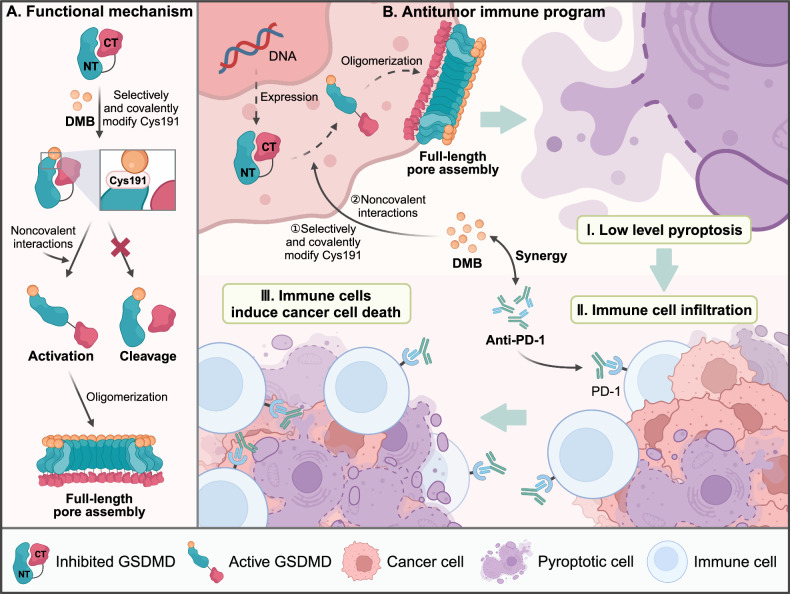
DMB: A Selective GSDMD Agonist for Inducing Pyroptosis and Enhancing Anti-Tumor Immunity. DMB is a direct and selective small molecule agonist of GSDMD that induces tumor regression and enhances anti-tumor immunity. It achieves this by covalently modifying the Cys191 residue in GSDMD, disrupting the interaction between the N-terminus (NT) and C-terminus (CT) (**A**). This keeps GSDMD in an activated state without cleavage, promoting pore formation and inducing pyroptosis while preserving the overall GSDMD structure. Additionally, non-covalent interactions between DMB and GSDMD further facilitate GSDMD activation. The anti-tumor efficacy of DMB depends on GSDMD expression in cancer cells, where it enhances immune responses, particularly by mobilizing lymphocytes, to selectively target cancer cells with minimal direct cytotoxicity. Moreover, DMB exhibits synergistic anti-cancer effects when combined with anti-PD-1 antibodies, offering a promising therapeutic approach for cancer treatment (**B**).

Immune checkpoint inhibitors (e.g., PD-1/PD-L1 inhibitors) have transformed cancer treatment in recent years [2]. However, resistance—either primary or acquired—affects many patients due to alterations in the tumor microenvironment [3]. Tumors are classified as “hot” or “cold” based on the level of immune cell infiltration. “Hot tumors” show high PD-L1 expression and abundant immune infiltration, leading to better responses to immunotherapy. Conversely, “cold tumors” have limited immune infiltration and suppressive cells, which diminish therapeutic efficacy [4]. Emerging evidence suggests that inducing pyroptosis can improve the immune microenvironment, transforming “cold” tumors into “hot” tumors, thereby enhancing the effectiveness of immune checkpoint inhibitors [5].

## The Role of Gasdermin D in Pyroptosis

As a key executor of pyroptosis, the gasdermin family, particularly GSDMD, plays a pivotal role in anti-tumor immunity. Pyroptosis is a type of programmed cell death mediated by inflammasomes, activated primarily through two pathways: canonical and non-canonical [6]. In the canonical pathway, intracellular pattern recognition receptors (such as NLRP3) detect infection or damage signals and assemble into inflammasomes, which subsequently activate pro-caspase-1, leading to the formation of active caspase-1. Activated caspase-1 cleaves GSDMD, releasing its N-terminal pore-forming domain (PFD), which forms pores in the cell membrane, resulting in disrupted substance exchange, cell swelling, and eventual rupture. In the non-canonical pathway, intracellular lipopolysaccharide (LPS) directly activates caspase-4/5 (in humans) or caspase-11 (in mice), which also cleaves GSDMD to form pores, triggering pyroptosis. Studies have demonstrated that GSDM activation in cancer cells can significantly improve immune responses [7, 8]. Consequently, targeted strategies that activate GSDM, particularly GSDMD, are now being explored for cancer treatment. However, a major challenge remains: how to effectively activate endogenous GSDMD in cancer cells.

To address this, Professor Hao Wu’s team investigated the structural dynamics of full-length GSDMD. They found that disrupting the interaction between GSDMD’s N-terminus (NT) and C-terminus (CT) triggers its activation without the need for inflammatory caspase cleavage. This breakthrough demonstrated that full-length GSDMD can form membrane pores directly, enabling pyroptosis. Through high-throughput screening, the team identified DMB as a potent GSDMD agonist. DMB binds strongly to GSDMD, triggering liposome leakage and directly activating pyroptosis.

## Mechanistic Validation of DMB in Cancer Models

To validate DMB’s mechanism of action, the researchers used CRISPR-Cas9 to generate GSDMD-altered triple-negative breast cancer (EMT6) and colorectal cancer (CT26) cell lines. DMB induced GSDMD-dependent pyroptosis, evidenced by cell death markers such as SYTOX Green staining and LDH/ATP release. In GSDMD- or GSDMD/GSDME-deficient cells, DMB-induced pyroptosis was significantly reduced, confirming GSDMD’s central role in the mechanism. Importantly, DMB’s effect was independent of caspase cleavage, signaling a novel pyroptotic pathway.

In vivo studies using wild-type and GSDMD-deficient mice further substantiated DMB’s anti-tumor effects, which were dependent on GSDMD expression. DMB substantially inhibited tumor growth in wild-type mice, but not in GSDMD-deficient models. Additionally, its efficacy was heightened in immune-competent mice, underscoring the immune system’s critical role in DMB’s mechanism.

Additionally, the authors’ team has determined through mass spectrometry analysis, structure-activity relationship studies, and mutation analysis that several critical features of DMB’s molecular structure are essential for activating GSDMD: (1) The methylsulfonyl (-SO_2_CH_3_) group at position 2 reacts with the sulfhydryl (-SH) group at Cys191 in GSDMD, forming a new covalent bond that disrupts the interaction between the N-terminal and C-terminal domains of GSDMD. This mechanism mimics the palmitoylation modification of GSDMD during inflammasome activation, where the palmitoylation of Cys191 is crucial for the pore-forming activity of GSDMD. (2) The chlorine substituents at carbon positions 6 and 7 of DMB significantly influence its electron density and hydrophobicity. These physicochemical properties are vital for the binding affinity and selectivity of DMB for GSDMD. Moreover, these groups may participate in non-covalent interactions with specific amino acid residues of GSDMD, such as hydrogen bonds, π-π stacking, or van der Waals forces, further stabilizing the complex between DMB and GSDMD and promoting GSDMD activation.

## Clinical Application and Future Directions

The therapeutic potential of DMB was explored in a B16 melanoma model expressing human GSDMD. DMB significantly inhibited tumor growth, increased survival rates, and enhanced immune cell infiltration in treated mice. Moreover, low-dose DMB combined with anti-PD-1 antibodies further augmented the anti-tumor effect, paving the way for future clinical applications.

Despite these promising findings, several key questions remain. First, DMB’s selectivity and specificity must be rigorously tested to rule out nonspecific effects on non-GSDMD pathways. Extensive screening and toxicological studies are necessary to confirm DMB’s specificity. Second, while GSDMD-deficient models validated DMB’s efficacy, broader testing across various tumor types and physiological conditions is essential. Humanized models and additional tumor variants should be considered to ensure the broad applicability of these findings.

The interaction between DMB and the immune system, particularly its impact on different immune cell types, warrants further investigation. Flow cytometry and immunohistochemistry can offer insights into immune cell lineage changes post-DMB treatment, helping clarify its immunomodulatory mechanisms. Lastly, detailed pharmacokinetic and toxicity studies, along with clinical trials, are critical for optimizing dosage, administration, and combination therapies.

## Conclusion

DMB represents a groundbreaking approach to cancer treatment by activating GSDMD-mediated pyroptosis and enhancing anti-tumor immunity. Its combination with immune checkpoint inhibitors shows great promise, but further studies are needed to address selectivity, immune system interactions, and clinical applicability. This research lays a solid foundation for future clinical trials and the development of novel anti-cancer therapies.
